# Prognostic, Clinicopathological, and Function of Key Cuproptosis Regulator *FDX1* in Clear Cell Renal Cell Carcinoma

**DOI:** 10.3390/genes13101725

**Published:** 2022-09-26

**Authors:** Song Zeng, He Zhang, Di Zhang, Xiaopeng Hu, Liming Song

**Affiliations:** 1 Department of Urology, Beijing Chao-Yang Hospital, Capital Medical University, Beijing 100020, China; 2 Institute of Urology, Capital Medical University, Beijing 100020, China; 3 Beijing Chao-Yang Hospital, Capital Medical University, Beijing 100020, China

**Keywords:** clear cell renal cell carcinoma, *FDX1*, cuproptosis, biomarker, prognosis

## Abstract

Clear cell renal cell carcinoma (ccRCC) is the most common histological subtype of renal cancer. Cuproptosis is suggested to be a novel therapy target for cancer treatment. However, the function of cuproptosis and its key regulator *FDX1* in ccRCC remains unclear. In this study, we adequately explored the prognostic factors, clinicopathological characteristics, and function of *FDX1* in ccRCC. We found that the expression of *FDX1* was significantly downregulated in ccRCC samples. Patients with a higher *FDX1* expression had a significantly better prognosis, including overall survival (OS) (Hazard ratio (HR): 2.54, 95% confidence interval (CI): 1.82–3.53, *p* < 0.001), disease-specific survival (DSS) (HR: 3.04, 95% CI: 2.04–4.54, *p* < 0.001), and progression-free survival (PFS) (HR: 2.54, 95% CI: 1.82–3.53, *p* < 0.001). *FDX1* was a clinical predictor to stratify patients into the high or low risk of poor survival, independent of conventional clinical features, with the area under the ROC curve (AUC) of 0.658, 0.677, and 0.656 for predicting the 5-year OS, DSS, and PFS. The nomogram model based on *FDX1* had greater predictive power than other individual prognostic parameters. *FDX1* mainly participated in the oxidative-related process and mitochondrial respiration-related processes but was not associated with immune infiltration levels. In conclusion, the cuproptosis key regulator *FDX1* could serve as a potential novel prognostic biomarker for ccRCC patients.

## 1. Introduction

Renal cell carcinoma is one of the most common and lethal urologic cancer types worldwide, with an estimated 431,288 new cancer cases and 179,368 deaths in 2020 [1]. Clear cell renal cell carcinoma (ccRCC) is the most common histological subtype of renal cancer. In the absence of reliable biomarkers and effective therapies, ccRCC has a high mortality rate and lousy prognosis. Currently, many treatments have been commonly used in day-to-day medical practice, such as non-specific cancer immunotherapy, targeted therapy, and novel immunotherapy agents [2]. Despite substantial therapeutic advancement for ccRCC, patients with metastatic ccRCC may have potential disadvantages with oncological outcomes. Currently, the most commonly used metric to determine the prognosis for ccRCC is tumor node metastasis classification (TNM) staging. However, the TNM staging system is not satisfactory enough to predict outcomes in ccRCC patients. Thus, integrating multiple prognostic factors, including TNM, to improve predictive capability is critical for enhancing the ccRCC patient prognosis [3].

Cuproptosis is a newly identified type of cell death program induced by copper [4]. Tsvetkov et al. found that copper-dependent, regulated cell death is distinct from known death mechanisms and depends on mitochondrial respiration [4]. Moreover, *FDX1* and protein lipoylation are the critical regulators of cuproptosis, and *FDX1* could affect the intracellular copper level. Copper is an essential mineral nutrient for living organisms as it is fundamental in many biological processes [5]. In this blockbuster research, the investigators found that cuproptosis primarily depends on intracellular copper accumulation [4]. Copper accumulation is closely associated with cancer growth, including proliferation, metastasis, and angiogenesis [6]. In particular, copper levels are elevated in serum and tumor tissues from patients with different tumors, such as liver, breast, lung, prostate, gastric, and bladder cancers [6]. Thus, copper dysregulation plays a critical role in cancer’s genesis, severity, and progression. It could be a vulnerable target against cancer development and progression.

Previous studies have proved that copper metal-binding compounds have considerable potential for cancer treatment [7]. Despite the excellent results of copper ionophores in vitro and in vivo, clinical studies of the ionophores in patients with cancer were not successful, such as elesclomol [8]. However, it showed antitumor activity in patients with low plasma lactate dehydrogenase levels, revealing that cells undergoing mitochondrial respiration are particularly sensitive to copper ionophores. Future studies of copper ionophores using a biomarker-driven approach should therefore be considered. However, studies on the effect of copper in ccRCC have been rarely reported. Therefore, the role of cuproptosis and its key regulator *FDX1* in ccRCC deserves further exploration.

In this study, we comprehensively analyzed the expression of *FDX1*, its predictive ability, and biological function in ccRCC patients using The Cancer Genome Atlas (TCGA), Gene Expression Omnibus (GEO), TIMER, and Gene Expression Profiling Interactive Analysis (GEPIA) databases. Our results identified the potential prognostic values of *FDX1* for overall survival (OS), disease-specific survival (DSS), and progression-free survival (PFS) in ccRCC patients. Moreover, *FDX1* was an independent prognostic biomarker for OS. The unique *FDX1*-based clinical risk scoring system presented a better prediction value in ccRCC patients than individual prognostic predictors.

## 2. Materials and Methods

### 2.1. Data Source

The TCGA database (https://portal.gdc.cancer.gov/) (accessed on 30 April 2022) was used to retrieve gene expression files, DNA methylation files, copy number mutation (CNV) files, and the clinical information of ccRCC patients. We searched and downloaded gene expression files in the GSE40435 cohort from the GEO database (https://www.ncbi.nlm.nih.gov/gds/) (accessed on 30 April 2022). We integrated and analyzed RNA-Seq data of normal tissues from Genotype-Tissue Expression (GTEx) (https://commonfund.nih.gov/GTEx/) (accessed on 30 April 2022) using Gene Expression Profiling Interactive Analysis (GEPIA2) (http://gepia2.cancer-pku.cn/#index) (accessed on 30 April 2022) [9]. We also applied the GEPIA2 to analyze the prognostic of the cuproptosis-related genes in pan-cancers, including 33 tumor types.

The cuproptosis-related genes were selected from the research, which identified the cuproptosis as a new type of cell death program induced by copper [4]. Among the 13 genes, *SLC31A1* is the copper importer while *ATP7A* and *ATP7B* are the copper exporters; *FDX1*, *LIAS*, *LIPT1*, *DLD*, *DLAT*, *PDHA1*, and *PDHB* are the positive regulators, while *MTF1*, *GLS*, and *CDKN2A* are the negative regulators.

We fully complied with the data access policies of GEO, TCGA, and GTEx when downloading the data in this study.

### 2.2. Evaluation of Cuproptosis-Related Genes Expression

To confirm the differential expression pattern of the cuproptosis-related genes, a total of 533 ccRCC samples and 72 adjacent normal renal samples were subjected to expression analysis by using the University of ALabama at Birmingham CANcer data analysis Portal (UALCAN) (http://ualcan.path.uab.edu/analysis.html) (accessed on 30 April 2022) [10]. Then a heatmap was drawn to visualize the hierarchical clustering analysis of the expression pattern of the cuproptosis-related genes.

### 2.3. Evaluation of FDX1 Expression and Prognostic Predictive Power

A total of three databases (TCGA, GTEx, and GSE40435) were used to validate the differential expression of *FDX1* between ccRCC samples and adjacent normal samples. The correlation among gender, age, pathological tumor stage, histological tumor grade, VHL mutation, PBRM1 mutation, and *FDX1* expression was investigated in the TCGA cohort. Furthermore, we applied the Tumor Immune Estimation Resource (TIMER) tool (https://cistrome.shinyapps.io/timer/) (accessed on 30 April 2022) to analyze the expression of *FDX1* in pan-cancers between tumor samples and adjacent normal samples [11].

To identify the factors that may significantly change the gene expression level of *FDX1*, we performed DNA methylation analysis and CNV analysis. The Spearman correlation test was performed to evaluate the correlation between DNA methylation level and gene expression level of *FDX1*. Wilcox test was used to analyze the significant difference in DNA methylation levels between ccRCC and normal renal samples. The expression levels of *FDX1* among different CNV groups (shallow deletion, diploid, gain) were compared using the Kruskal–Wallis test. Values were considered to be significantly different when the P value was less than 0.05.

To investigate the prognostic performance of *FDX1* in ccRCC, we firstly applied the X-tile 3.6.1 software to select the best cut-off value to classify the ccRCC patients into low- and high-expression groups [12]. Then, the Kaplan–Meier (KM) survival analysis and time-dependent receiver operating characteristic (ROC) curves were performed to evaluate the ability of *FDX1* to predict OS, DSS and PFS by utilizing the survminer and survivalROC R package, respectively. A total of 530, 519, and 530 ccRCC patients were enrolled in the OS, DSS, and PFS analysis, respectively.

### 2.4. Independence of the FDX1 from Clinical Features and Nomogram Construction

A total of 519 ccRCC patients in the TCGA cohort with *FDX1* expression data, survival time, age, gender, pathological tumor stage, and histological tumor grade, were subjected to subsequent analysis, to assess the independent prognostic ability of *FDX1* for ccRCC patients. The forest plot was conducted utilizing the ggplot2 R package to visualize the predictive ability better. The univariate and multivariate Cox regression analysis was conducted using the survival R package.

To further predict the survival rates of ccRCC patients at 1, 5, and 10 years in a clinical setting, we constructed a novel nomogram containing clinical features and *FDX1* based on the multivariate Cox analysis results utilizing the rms R package. The time-dependent ROC curves were plotted to evaluate the nomogram’s predictive performance. The concordance index (C-index) was used to evaluate the nomogram’s discriminant ability, and it was corrected by a bootstrap method with 1000 resamples. Moreover, the calibration curves were utilized to evaluate the agreement between model-predicted and actual risks [13]. Decision curve analysis (DCA) was used to assess the nomogram’s clinical utility potential.

### 2.5. Gene Set Enrichment Analysis (GSEA)

GSEA was performed based on gene correlations to investigate the potential biological function of *FDX1* [14]. An annotated gene set file (c5.bp.v7.0.entrez.gmt) was chosen as the reference gene set. The threshold was set at levels of |NES| > 2 and *p* < 0.01.

### 2.6. The Immune Landscape of the FDX1

To further study the relationship between *FDX1* and tumor microenvironment, we analyzed the correlation of *FDX1* and major immune cells using TIMER, a comprehensive resource for systematical analysis of immune infiltrates across diverse cancer types [11]. Moreover, the TIMER2.0 version could provide immune infiltrate abundance estimates using multiple immune cell deconvolution methods, which can be cross-validated.

### 2.7. Statistical Analysis

R software (version 4.1.2) was conducted to analyze data. Univariate and multivariate Cox analysis was performed with the R package “survival” [15]. The R package “survival ROC” was used to evaluate the prognostic role of K-M curves and time-dependent receiver operating characteristics [16]. Additionally, stratification analysis was conducted based on the clinical characteristics of different subgroups. The R “pheatmap” package was performed to generate the heatmap plot. The R package ‘rms’ was applied to plot nomograms and calibration curves [17]. DCA was used to analyze the clinical benefits with the R package ‘ggDCA’. Categorical variables were presented as counts (percentages), while continuous variables were presented as median with interquartile range (IQR). Kruskal−Wallis and Wilcoxon test were used to compare differences between groups. This study considered the *p*-value < 0.05 as statistically significant.

## 3. Results

### 3.1. Differential Expression of Cuproptosis-Related Genes in ccRCC and Its Prognostic Relevance in Pan-Cancers

**Figure 1:**
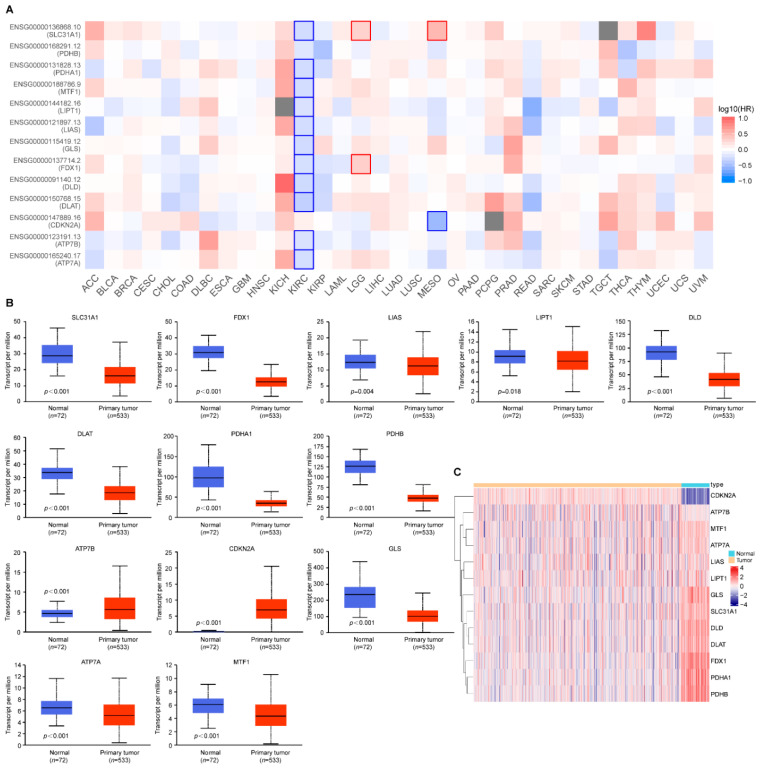
Cuproptosis-related gene expression in ccRCC and its prognostic relevance in pan-cancers. (**A**) The prognostic relevance of cuproptosis-related genes in pan-cancers; (**B**) The expression pattern of cuproptosis-related genes in ccRCC; (**C**) Heatmap plots of cuproptosis-related genes between ccRCC samples and adjacent normal samples. Red denotes upregulated genes, and blue denotes downregulated genes in heatmaps. The horizontal axis of the heatmaps represents the samples. *p* values less than 0.05 were considered to be statistically significant.

To investigate the prognostic relevance of the cuproptosis-related genes, we applied GEPIA2 to analyze its prognostic value in pan-cancers. It is worth noting that, as shown in Figure 1A, the low expression of 11 cuproptosis-related genes, except *PDHB* and *CDKN2A*, was associated with poor prognosis in ccRCC tumors. Therefore, we explored the differential expression pattern of the cuproptosis-related genes in ccRCC patients by utilizing UALCAN. The expression of the copper importer (*SLC31A1*) and all the positive regulators of cuproptosis (*FDX1*, *LIAS*, *LIPT1*, *DLD*, *DLAT*, *PDHA1*, and *PDHB*) was significantly downregulated in the ccRCC samples compared with the normal renal samples (Figure 1B). However, the expression of the copper exporters (*ATP7B*) and the negative regulators (*CDKN2A*) was significantly upregulated in the ccRCC samples compared with the normal renal samples. The heat-map of the cuproptosis-related genes in ccRCC samples and adjacent normal renal samples is shown in Figure 1C.

### 3.2. The Key Regulator Cuproptosis FDX1 Is Downregulated in ccRCC

**Figure 2:**
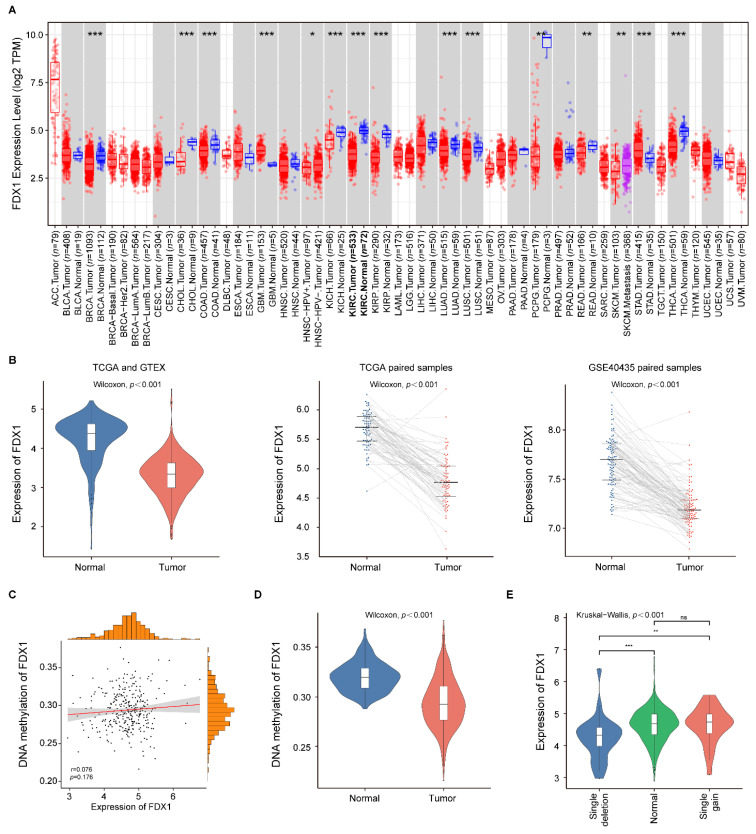
*FDX1* expression in pan-cancers and ccRCC patients. (**A**) The expression of *FDX1* in different tumor samples compared with normal samples; (**B**) *FDX1* expression was significantly decreased in ccRCC samples compared with normal renal samples in TCGA, GTEx, and GSE40435 cohorts; (**C**) Correlation analysis of *FDX1* gene methylation and expression levels; (**D**) The methylation level of *FDX1* in the ccRCC samples was significantly lower than in the adjacent normal samples; (**E**) Expression patterns of *FDX1* among different CNV groups. *p* values less than 0.05 were considered to be statistically significant and ns represents no significant differences. * *p* < 0.05, ** *p* < 0.01, *** *p* < 0.001.

The expression analysis of *FDX1* in pan-cancers using the TIMER database showed that *FDX1* mRNA expression was significantly lower in BRCA (breast invasive carcinoma), CHOL (cholangiocarcinoma), COAD (colon adenocarcinoma), ccRCC, KICH (kidney chromophobe), KIRP (kidney renal papillary carcinoma), LUAD (lung adenocarcinoma), LUSC (lung squamous cell carcinoma), PCPG (Pheochromocytoma and Paraganglioma), READ (rectum adenocarcinoma), and THCA (thyroid carcinoma) samples compared with the corresponding normal samples (Figure 2A). Only GBM (Glioblastoma multiforme) and STAD (stomach adenocarcinoma) samples showed higher *FDX1* expression than the corresponding normal samples. These results indicated that *FDX1* expression was downregulated in the majority of tumors.

To further evaluate the differential expression level of *FDX1* expression in ccRCC patients, we compared the expression level of the ccRCC samples and normal renal samples in three databases (TCGA, GTEx, and GSE40435). As indicated in Figure 2B, the *FDX1* expression was consistently downregulated in the ccRCC samples compared with the normal renal samples through all datasets. Then, we investigated the *FDX1* methylation status of the ccRCC samples, and we did not find a significant correlation between the *FDX1* expression level and its methylation status in the TCGA cohort (r = 0.076, *p* = 0.176; Figure 2C). Nevertheless, the methylation level of *FDX1* in the ccRCC samples was significantly lower than in the normal renal samples (Figure 2D). Subsequently, we evaluated the effect of CNVs on *FDX1* expression in ccRCC samples. The result showed a significant difference in *FDX1* expression among the single deletion group, normal group, and single gain group (Figure 2E). Thus, the expression level of *FDX1* might be changed by the single deletion in ccRCC.

### 3.3. Correlation between FDX1 Expression and Clinical Features

**Figure 3:**
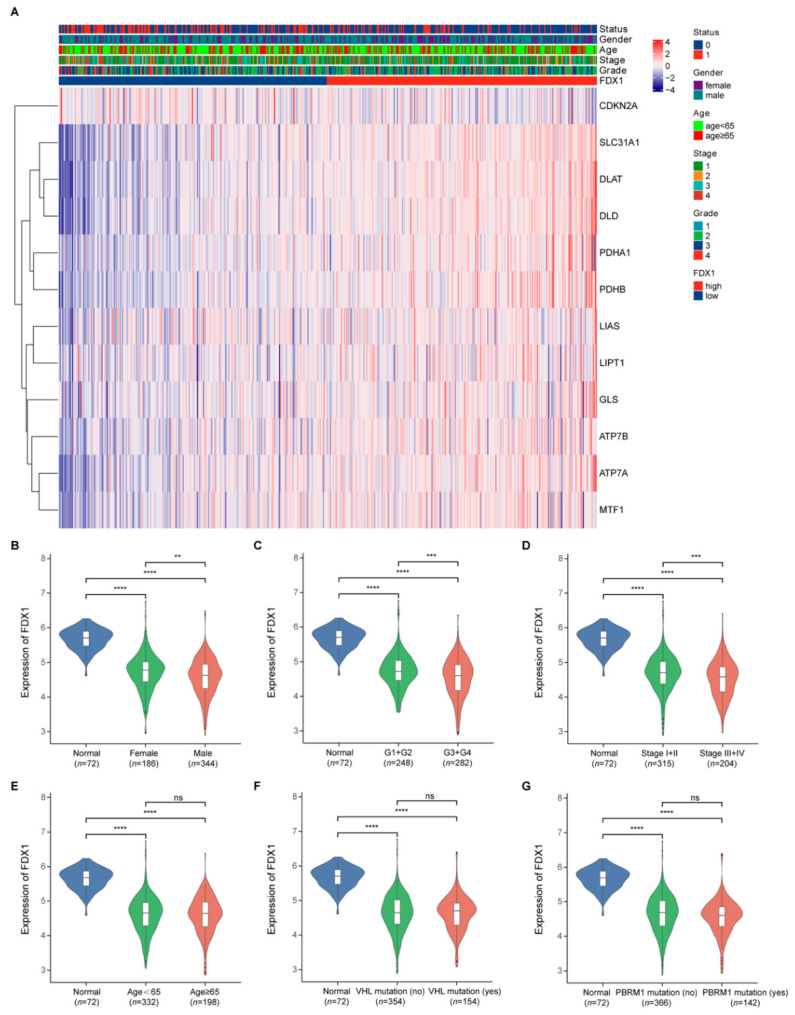
*FDX1* expression in different subgroup ccRCC patients. (**A**) The correlation between the *FDX1* expression and the clinical features; (**B**–**G**) *FDX1* expression in ccRCC patients according to different clinical characteristics. *p* values less than 0.05 were considered to be statistically significant. ** *p <* 0.01, *** *p <* 0.001, **** *p <* 0.0001.

The expression level of *FDX1* in ccRCC samples with different ages, gender, pathological tumor stage, histological tumor grade, VHL mutation, and PBRM1 mutation was analyzed. VHL and PBRM1 mutations were the most frequently mutated genes in ccRCC [3]. The relationship between *FDX1* expression and the clinical characteristics of ccRCC patients is summarized in Table 1. It was determined that the *FDX1* expression had strong correlations with gender (*p* = 0.006), histological tumor grade (*p* < 0.001), and pathological tumor stage (*p* < 0.001). However, we did not observe a correlation between *FDX1* expression and age (*p* = 0.816), VHL mutation (*p* = 0.967), or PBRM1 mutation (*p* = 0.136). Furthermore, we visualized the correlation between the *FDX1* expression and the clinical features in the form of a heatmap (Figure 3A).

As indicated in Figure 3, the *FDX1* expression was downregulated in ccRCC samples in all the subgroup ccRCC patients compared with that in the normal renal samples. Moreover, in ccRCC samples, *FDX1* showed a higher expression in female patients than in male patients (Figure 3B); a higher expression in patients with lower histological tumor grade (G1 + G2) than in patients with higher histological tumor grade (G3 + G4) (Figure 3C); and a higher expression in patients with lower pathological tumor stage (Stage I + II) than in patients with higher pathological tumor stage (Stage III + IV) (Figure 3D). However, we observed no significant differences in *FDX1* expression in the age (Figure 3E), VHL mutation (Figure 3F), and PBRM1 mutation (Figure 3G) subgroups.

### 3.4. The Prognostic Predictive Power of FDX1 Expression in ccRCC

**Figure 4:**
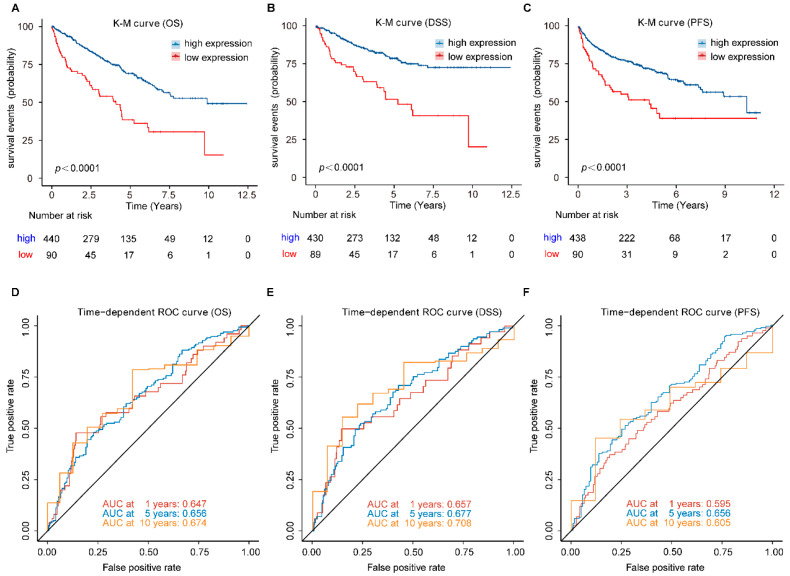
Prognostic analysis of *FDX1* in ccRCC patients. (**A**) OS, (**B**) DSS, and (**C**) PFS, was significantly higher in the high-expression group than in the low-expression group in ccRCC patients; (**D**–**F**) Time-dependent ROC curve analysis of *FDX1*. OS, overall survival; DSS, disease-specific survival; PFS, progression-free survival.

To illustrate the prognostic ability of *FDX1* in patients with ccRCC, we conducted a pooled analysis to evaluate its power in predicting OS, DSS, and PFS. We first calculated the best cut-off value of *FDX1* expression, and patients with expression levels above 4.15 were divided into the high-expression group, while those with expression levels below or equal to 4.15 were classified into the low-expression group. The K-M survival results revealed that high expression of *FDX1* was associated with better prognosis, including OS (Hazard ratio (HR): 2.54, 95% confidence interval (CI): 1.82–3.53, *p* < 0.001), DSS (HR: 3.04, 95% CI: 2.04–4.54, *p* < 0.001), and PFS (HR: 2.54, 95% CI: 1.82–3.53, *p* < 0.001) (Figure 4A–C). The time-dependent ROC curve results further confirmed the stable prognostic value of the *FDX1*. The area under the ROC curve (AUC) value of *FDX1* was 0.647 at 1 year, 0.658 at 5 years, and 0.674 at 10 years when predicting OS (Figure 4D); 0.657 at 1 year, 0.677 at 5 years, and 0.708 at 10 years when predicting DSS (Figure 4E); 0.595 at 1 year, 0.656 at 5 years, and 0.605 at 10 years when predicting PFS (Figure 4F).

### 3.5. Stratification Analysis of OS for the FDX1 in ccRCC Patients

**Figure 5:**
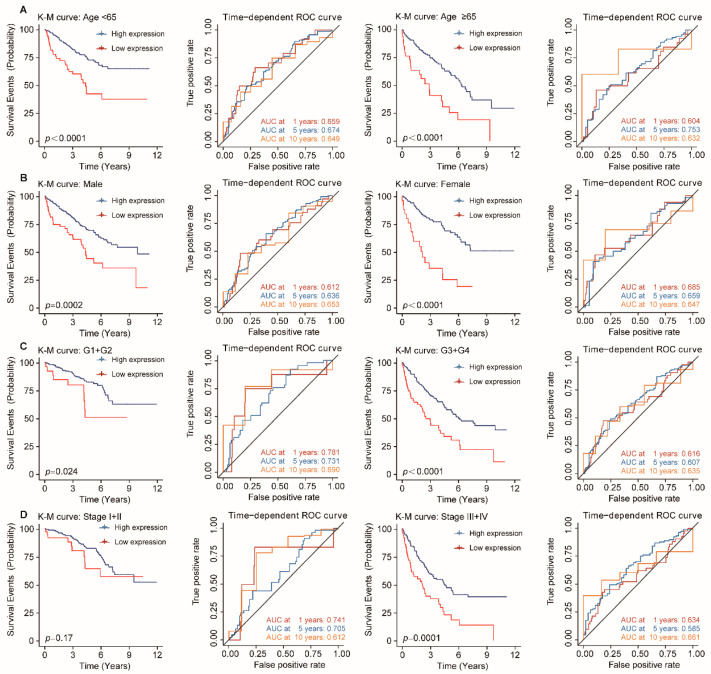
Stratification analysis of OS for *FDX1* in ccRCC patients. Kaplan–Meier and time-dependent ROC curves illustrate the prognostic value of *FDX1* based on the stratification of different clinical characteristics. Notes: (**A**) Age; (**B**) Gender; (**C**) Histological tumor grade; (**D**) Pathological tumor stage.

To further validate whether the predictive ability of *FDX1* would remain stable in different subgroups of ccRCC patients, we conducted the stratification analysis based on clinical characteristics. ccRCC patients were allocated to two groups according to age, gender, histological tumor grade, and pathological tumor stage. The results of the relationship between *FDX1* expression and prognosis in ccRCC with different clinical factors by the Kaplan–Meier plotter are summarized in Table 2. As shown in Figure 5, patients in the high-expression group showed better survival than those in the low-expression group with younger or older patients, male or female patients, grade low or grade high tumors, stage I and II or stage III and IV tumors. Therefore, *FDX1* still had powerful and stable prognostic predictive power for ccRCC patients in distinct subgroups.

### 3.6. FDX1 Is Independent of Traditional Clinical Characteristics for ccRCC Patients

**Figure 6:**
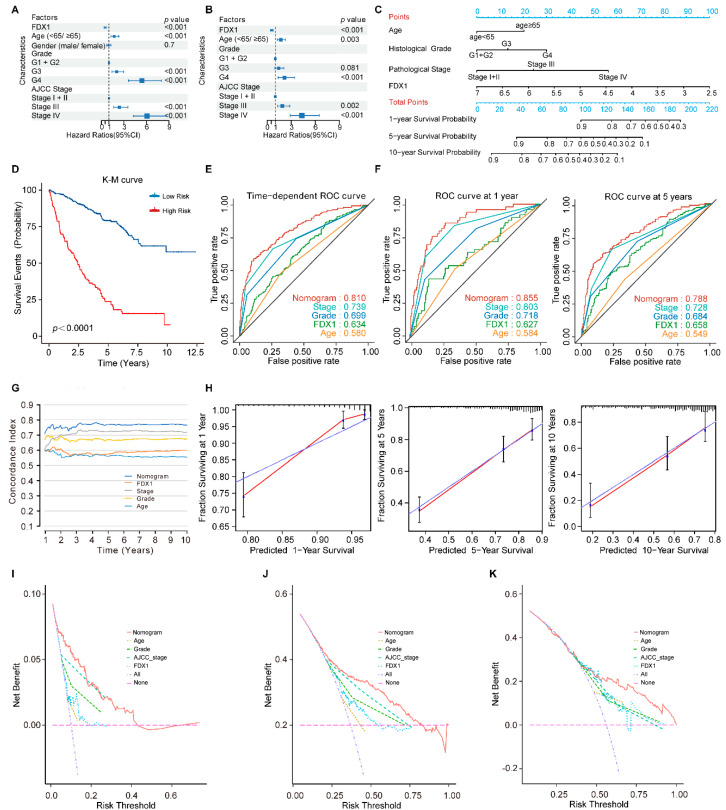
The *FDX1*-based nomogram model in ccRCC patients. (**A**) Univariate and (**B**) multivariate regression analysis of the relation between *FDX1* and clinical features regarding prognostic value; (**C**) Nomogram for predicting the probability of 1−, 5−, and 10−year OS for ccRCC patients; (**D**) Kaplan–Meier curves demonstrate the prognostic performance of the nomogram; (**E**,**F**) Time-dependent ROC curves of the nomogram; (**G**) The prognostic performance was compared among the nomogram, *FDX1*, and conventional clinical characteristics by calculating the C-index; (**H**) Calibration plots of the nomogram for predicting the probability of OS at 1, 5, and 10 years; (**I**–**K**) DCA plots of the nomogram, *FDX1*, and clinical characteristics for predicting the probability of OS at 1, 5, and 10 years.

To identify whether *FDX1* is an independent clinical predictor for OS in ccRCC patients, clinical characteristics, including age, gender, histological tumor grade, and pathological tumor stage, were adjusted by univariate and multivariate Cox regression analysis. The result was summarized in Table 3. The results of the univariate analysis indicated that *FDX1* was significantly associated with OS (HR: 0.489, 95% CI: 0.372–0.639, *p* < 0.001; Figure 6A). As shown in Figure 6B, using multivariate analysis, *FDX1* remained an independent predictor with an HR of 0.562 (95% CI: 0.422–0.749, *p* < 0.001).

### 3.7. Development and Validation of an FDX1-Based Nomogram Model

Previous studies have demonstrated that the nomogram evaluation model can predict a cancer patient’s prognosis more accurately [13,18]. Therefore, we developed a nomogram model to predict survival probability rates by combining *FDX1* expression and independent clinical prognostic factors (age, histological tumor grade, and pathological tumor stage) (Figure 6C).

By calculating the total model points of each ccRCC patient, we stratified the patients into high- and low-risk subgroups based on the optimal cut-off point of 107 (high-risk group: ≥107, low-risk group: <107). Compared to the low-risk patients, the high-risk patients turned out to suffer significantly shorter OS, with a 5.95-fold higher risk (95% CI: 4.38–8.07, *p* < 0.001; Figure 6D). The time-dependent ROC curve analysis revealed that the nomogram model had greater predictive power than other predictors (Figure 6E,F). The mean C-index of the nomogram model was higher (0.766) than other predictors (0.557 to 0.717) (Figure 6G). Moreover, the calibration plots indicated a good agreement between the actual and estimated probabilities at 1, 5, and 10 years, with lines close to 45 degrees (Figure 6H). DCA was a new approach to evaluating prediction models. We performed the DCA analysis to compare the clinical predictive value of the nomogram model and other individual predictors [19]. The results showed that the nomogram model had a better net benefit and broader threshold probability, implying it had the best clinical utility compared with other independent predictors (Figure 6I–K).

### 3.8. Functional Enrichment Analysis and Immune Cells Infiltration Analysis

**Figure 7:**
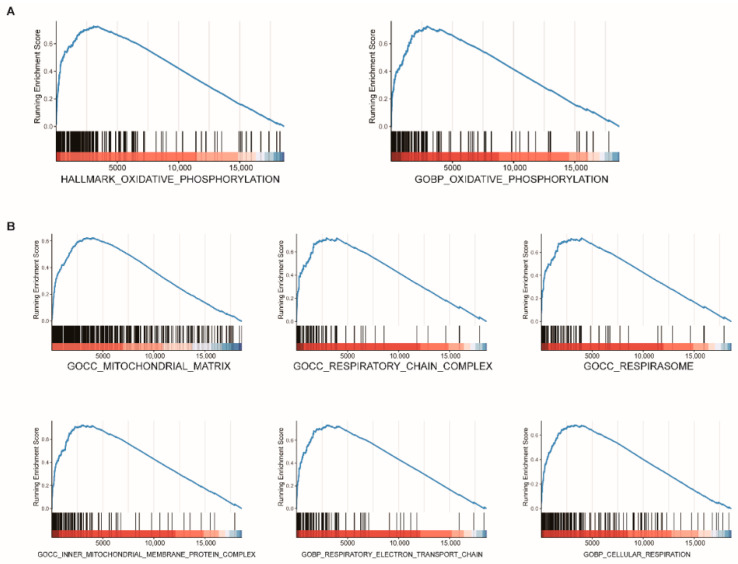
Functional enrichment analysis of *FDX1* in ccRCC. GSEA plot reveals that *FDX1* mainly participated in oxidative-related processes (**A**) and mitochondrial respiration-related processes (**B**).

GSEA was conducted to elucidate further the underlying biological functions of *FDX1* based on gene correlations. The results revealed that *FDX1* mainly participated in the oxidative-related process, such as OXIDATIVE PHOSPHORYLATION (Figure 7A), mitochondrial respiration-related processes, such as MITOCHONDRIAL MATRIX, RESPIRATORY CHAIN COMPLEX, RESPIRASOME, INNER MITOCHONDRIAL MEMBRANE PROTEIN COMPLEX, RESPIRATORY ELECTRON TRANSPORT CHAIN, and CELLULAR RESPIRATION (Figure 7B). To further analyze the correlation between the immune cell infiltration and *FDX1*, we utilized TIMER2.0 and found that no significant correlations were observed between the *FDX1* expression and the ccRCC tumor microenvironment (Supplementary Figure S1).

## 4. Discussion

As the most common malignant solid lesion within the kidney in adults, renal cell carcinoma accounts for approximately 90% of all renal malignancies, and ccRCC is the most common histological subtype of renal cell carcinoma [20]. Despite the availability of multiple therapeutic methods for patients with ccRCC, its high recurrence rate and the unsatisfactory long-term prognosis of patients with metastatic ccRCC lead to an increasing demand to search for novel prognostic markers and effective therapeutic targets to improve the prognosis of ccRCC patients. The cuproptosis, a copper-induced cell death, reported by Tsvetkov et al. suggests its vital role in developing multiple cancers and using copper to treat cancer [21]. Few studies have explored the specific effect and prognostic value of cuproptosis in ccRCC, especially its key regulator *FDX1*.

In this study, we found that all the cuproptosis-related genes were differentially expressed between ccRCC samples and normal renal samples, and the low expression of most cuproptosis-related genes was significantly associated with poor prognosis. We also verified that *FDX1* was an independent, powerful, and stable clinical predictor for OS in ccRCC patients. The function analysis revealed that *FDX1* mainly participated in the oxidative-related process and mitochondrial respiration-related processes. The nomogram consisting of *FDX1* and other clinical prognostic factors can accurately predict the prognosis of ccRCC patients and assist the clinicians in risk assessment for individual ccRCC patients.

Copper is a mineral nutrient increasingly implicated in cell proliferation and death pathways [22]. The total pool of intracellular copper is divided into two subsets: a tightly bound protein pool and a bioavailable labile pool [23,24]. Previous studies have revealed the key molecular pathways that regulate copper acquisition, trafficking, storage, and export [25,26,27]. The copper importer (*SLC31A1*) and the copper exporters (*ATP7B*) are the key targets in mammalian copper homeostasis. In the present study, we found that *SLC31A1* was significantly downregulated while *ATP7B* was upregulated in the ccRCC samples, suggesting an imbalance of copper homeostasis in ccRCC tissues. The strong correlation between the low expression of the positive regulators of cuproptosis and poor prognosis further indicated the potential close associations between cuproptosis and ccRCC.

*FDX1* encodes a small iron-sulfur protein that participates in the reduction of mitochondrial cytochrome and reduces Cu^2+^ to its more toxic form, Cu^1+^ [28]. It is the key regulator of cuproptosis and an upstream regulator of protein lipoylation in the tricarboxylic acid (TCA) cycle [4]. The TCA cycle is an important biological process of mitochondrial respiration. Consistent with the finding that *FDX1* played an essential role in mitochondrial respiration, our study demonstrated that *FDX1* mainly participated in the oxidative-related process and mitochondrial respiration-related processes in ccRCC tissues. To explore the clinicopathology and prognostic factors of *FDX1* in ccRCC, we found that the *FDX1* expression was downregulated in ccRCC samples in all the subgroup ccRCC patients, which might be caused by the single deletion in ccRCC. This result corroborated the findings of previous studies in LUAD and ccRCC [29,30]. Subsequently, we revealed that the expression of *FDX1* was highly correlated with gender, histological tumor grade, and pathological tumor stage, thus revealing that low *FDX1* expression had a strong relationship with a malignant ccRCC phenotype. In addition, we found that *FDX1* was an independent predictor for OS in ccRCC patients after adjusting for other traditional clinical features. Hence, we constructed a nomogram model combining *FDX1* expression and independent clinical prognostic factors. The time-dependent ROC curve analysis, C-index, and DCA validated the strong prognostic value of the nomogram for ccRCC patients. The main advantage of this model resides in developing a unique *FDX1*-based clinical risk scoring system for ccRCC patients. All the above results confirmed that *FDX1* had the potential to be a novel prognostic biomarker for ccRCC patients.

Our study is the first to explore the prognostic factors, clinicopathological characteristics, and function of key cuproptosis regulator *FDX1* in ccRCC. An in-depth understanding of *FDX1*-regulated cuproptosis in KIRC will help enable a new way to kill cancer cells by exploiting the distinct action of copper.

However, this study still has several limitations that need to be improved in the future. First, we only validated the prognostic value of *FDX1* in the TCGA dataset, no clinical cohorts were available for further validation, which is urgently warranted in future research. Moreover, prospective studies are needed to confirm the predictive ability of *FDX1* for ccRCC. Second, although the unique *FDX1*-based clinical risk scoring system showed strong prognostic value in ccRCC patients, other significant cuproptosis-related genes with predictive values were not explored in this study. Third, a strong experimental basis is still lacking for further revealing the role of cuproptosis and *FDX1* in ccRCC development and progression. The exact molecular mechanism of *FDX1* in ccRCC needed more experimental research to be further explored.

## 5. Conclusions

In summary, this study systematically analyzed the expression patterns of cuproptosis-related genes and the prognostic factors, clinicopathological characteristics, and function of key cuproptosis regulator *FDX1* in ccRCC. The findings indicated that *FDX1* could serve as a potential novel prognostic biomarker for ccRCC patients. Additionally, we developed a unique *FDX1*-based clinical risk scoring system with strong prognostic value, and it may help clinicians better predict the prognosis of ccRCC patients.

## Figures and Tables

**Table 1:** The association between *FDX1* expression and clinical characteristics.

Characteristics	Total (N)	*FDX1* Expression	
		OR (95% CI)	*p* Value
Age (<65 vs. ≥65)	530	0.96 (0.69–1.34)	0.816
Gender (female vs. male)	530	1.62 (1.15–2.29)	0.006
Grade	530		
G1 + G2		Reference	
G3 + G4		0.48 (0.34–0.67)	<0.001
AJCC Stage	519		
Stage I + II		Reference	
Stage III + IV		0.57 (0.41–0.81)	<0.001
VHL_mutated (yes vs. no)	508	0.99 (0.7–1.41)	0.967
PBRM1_mutated (yes vs. no)	508	0.76 (0.53–1.09)	0.136

Abbreviations: OR, odds ratio; CI, confidence interval. *p* values less than 0.05 were considered to be statistically significant.

**Table 2:** Correlation of *FDX1* mRNA expression and prognosis in different subgroup ccRCC patients.

Characteristics	Overall Survival		
	Total (N)	HR (95% CI)	*p* Value
Age			
<65	332	2.58 (1.65–4.04)	<0.0001
≥65	198	2.66 (1.63–4.35)	<0.0001
Gender			
male	344	2.15 (1.42–3.24)	0.0002
female	186	3.7 (2.13–6.41)	<0.0001
Grade			
G1 + G2	248	2.28 (1.09–4.74)	0.024
G3 + G4	282	2.22 (1.53–3.22)	<0.0001
AJCC Stage			
Stage I + II	315	1.6 (0.81–3.17)	0.17
Stage III + IV	204	2.13 (1.44–3.15)	0.00011

Abbreviations: HR, hazard ratio; CI, confidence interval. *p* values less than 0.05 were considered to be statistically significant.

**Table 3:** Univariate and multivariate Cox regression analyses for predicting OS.

Factors	Univariate		Multivariate	
	HR (95% CI)	*p* Value	HR (95% CI)	*p* Value
*FDX1*	0.489 (0.372–0.639)	<0.001	0.562 (0.422–0.749)	<0.001
Age (<65/≥65)	1.714 (1.272–2.309)	<0.001	1.693 (1.252–2.289)	0.003
Gender (male/female)	0.941 (0.691–1.283)	0.7		
Grade				
G1 + G2	1		1	
G3	2.031 (1.398–2.95)	<0.001	1.414 (0.958–2.085)	0.081
G4	5.38 (3.617–8.002)	<0.001	2.174 (1.375–3.436)	<0.001
AJCC Stage				
Stage I + II	1		1	
Stage III	2.41 (1.641–3.539)	<0.001	1.884 (1.263–2.809)	0.002
Stage IV	6.073 (4.256–8.664)	<0.001	4.325 (2.878–6.501)	<0.001

Abbreviations: OS, overall survival; HR, hazard ratio; CI, confidence interval. *p* values less than 0.05 were considered to be statistically significant.

## Data Availability

All data generated or analyzed during this study are included in this published article and its Supplementary Materials.

## Supplementary Materials

The following supporting information can be downloaded at: https://www.mdpi.com/article/10.3390/genes13101725/s1. Supplementary Figure S1. The immune landscape of the *FDX1* in ccRCC. (A) Correlation analysis between *FDX1* expression and tumor purity based on TIMER; (B) Correlation analysis between *FDX1* expression and six immune cell infiltration in ccRCC with TIMER algorithm; (C) The relationship between *FDX1* expression and B cell; (D) The relationship between *FDX1* expression and CD8+T cell; (E) The relationship between *FDX1* expression and Macrophage; (F) The relationship between *FDX1* expression and Dendritic cell. *p* values less than 0.05 were considered to be statistically significant.
