# Supplementary material for: Prognostic, Clinicopathological, and Function of Key Cuproptosis Regulator FDX1 in Clear Cell Renal Cell Carcinoma
Source: Genes (Basel). 2022 Sep 26;13(10):1725. doi: 10.3390/genes13101725 (PMC9601362; doi:10.3390/genes13101725)
Supplement: Supplementary file 1 [file genes-13-01725-s001.zip › genes-1885265-Supplementary.pdf]

genes

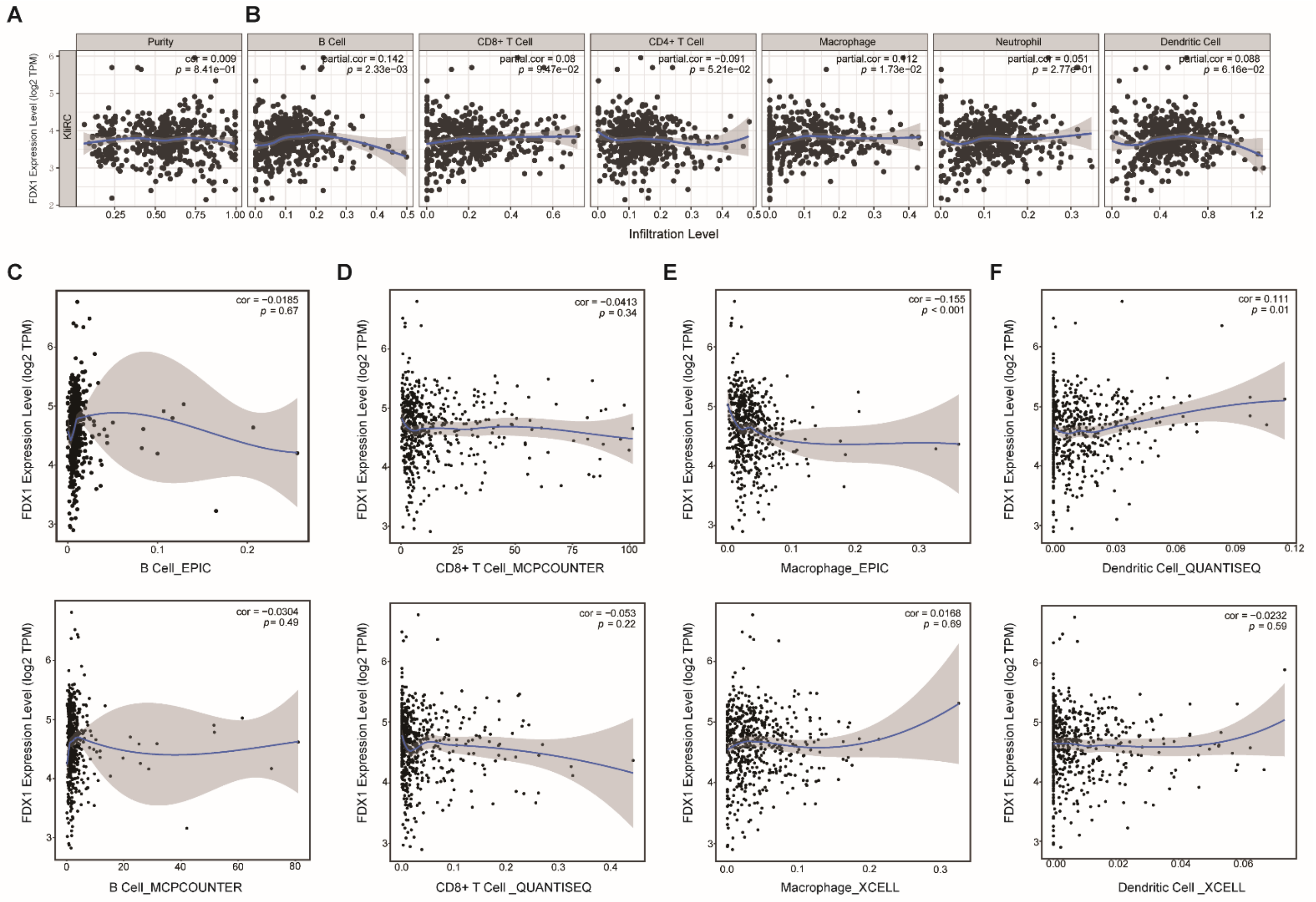

Supplemental Figure S1. The immune landscape of the *FDX1* in ccRCC. (A) Correlation analysis between *FDX1* expression and tumor purity based on TIMER; (B) Correlation analysis between *FDX1* expression and six immune cell infiltration in ccRCC with TIMER algorithm; (C) The relationship between *FDX1* expression and B cell; (D) The relationship between *FDX1* expression and CD8+T cell; (E) The relationship between *FDX1* expression and Macrophage; (F) The relationship between *FDX1* expression and Dendritic cell. *p* values less than 0.05 were considered to be statistically significant.
